# Outcomes of a Pharmacological Protocol with Pentoxifylline and Tocopherol for the Management of Medication-Related Osteonecrosis of the Jaws (MRONJ): A Randomized Study on 202 Osteoporosis Patients

**DOI:** 10.3390/jcm12144662

**Published:** 2023-07-13

**Authors:** Gianluca Colapinto, Funda Goker, Riccardo Nocini, Massimo Albanese, Pier Francesco Nocini, Salvatore Sembronio, Francesca Argenta, Massimo Robiony, Massimo Del Fabbro

**Affiliations:** 1 Indipendent Researcher, Chief Medical Officer, Oral Med Care srl, 70032 Bitonto, BA, Italy; 2 Department of Biomedical, Surgical and Dental Sciences, University of Milano, 20122 Milan, Italy; funda.goker@unimi.it (F.G.); argentafrancesca@gmail.com (F.A.); 3 Dental and Maxillo-Facial Surgery Unit, Fondazione IRCCS Ca’ Granda Ospedale Maggiore Policlinico di Milano, 20122 Milan, Italy; 4 Dipartimento Scienze Chirurgiche Odontostomatologiche e Materno-Infantili, Università degli Studi di Verona, 37134 Verona, Italy; riccardo.nocini@univr.it (R.N.); massimo.albanese@univr.it (M.A.); 5 Dental and Maxillofacial Surgery Clinics, University of Verona, 37134 Verona, Italy; pierfrancesco.nocini@univr.it; 6 Department of Medical Area, University of Udine, 33100 Udine, Italy; salvatore.sembronio@uniud.it (S.S.); massimo.robiony@uniud.it (M.R.)

**Keywords:** osteonecrosis, pentoxifylline, tocopherol, BRONJ, MRONJ, tooth extraction, sequestrectomy

## Abstract

Medication-related osteonecrosis of the jaws (MRONJ) is a challenging situation in clinics. Previous studies have shown that pentoxifylline combined with tocopherol proved to be beneficial in patients with osteoradionecrosis, due to their antioxidant and antifibrotic properties. The aim of this randomized study was to evaluate the effect of pentoxifylline and tocopherol in patients that had developed MRONJ after tooth extractions. The study population consisted of 202 Stage I MRONJ female patients with an average age of 66.4 ± 8.3 years, who were divided into two groups. The test group (n = 108) received a pharmacological protocol with pentoxifylline and tocopherol (2 months pre-operatively and 6 months post-operatively). The control group (n = 94) had sequestrectomy operations without any pharmacological preparation. The main outcomes were clinical healing of the mucosa after 1 month, and clinical and radiographic healing of the bone lesion at 6 months. In the test group all patients had mucosal healing and there was only one relapse within 6 months. In the control group, in 17% of the patients the mucosa did not heal, 71% of the patients relapsed within two months, and 7% developed infectious complications (such as abscess or phlegmon). After 6 months, the control group patients with persisting issues were prescribed pentoxifylline and tocopherol, as in the test group. At a subsequent follow-up, all those patients healed completely. Patients were monitored for a period of 7.8 ± 0.3 years, during which no relapse or additional problems were reported. As a conclusion, pentoxifylline and tocopherol protocol seems to be beneficial in the management of MRONJ patients.

## 1. Introduction

Osteonecrosis (avascular necrosis) is caused by an alteration of the physiological bone metabolism during the bone healing process, which may lead to bone death [1,2]. Osteonecrosis of the jaw (ONJ) is a relatively rare but critical condition manifested by necrotic bone lesions localized in the maxilla and/or in the mandible. ONJ has been linked to the use of potent antiresorptive and antiangiogenic agents and has been termed medication related osteonecrosis of the jaw (MRONJ) [3]. Various drugs with different mechanisms of action and effects can potentially trigger MRONJ [4,5,6]. Among these drugs, the most frequently implicated in MRONJ are bisphosphonates (BPs). Currently, BPs are the most famous and major class of drugs for the treatment of bone diseases such as osteoporosis [7]. BPs can specifically block the activity of osteoclasts and therefore bone resorption, with a different mechanism of action and different anti-resorptive efficacy power which is related to their chemical structure [1,8]. The BPs are divided into two large categories, amino-BPs and non-amino-BPs. The former are considered to be more powerful and effective in counteracting bone resorption, as compared to non-amino BPs. However, they are also associated with higher risks of MRONJ [9].

The first early objective sign of an increase in bone density due to the antiresorptive action of BPs is the sclerotization of the alveolar lamina with a reduction in the space of the periodontal ligament that can be targeted by an intraoral radiograph [10]. This increase in density can extend into the context of the entire alveolar process of the maxilla which results in a reduction of the trabecular space with vascular depletion and reduced capillary function, with the attempt to develop new vessels with a diameter slightly less than that of red blood cells. This determines a condition of chronic ischemia and the possibility of avascular necrosis of the bone itself [10,11,12,13]. Based on this pathogenetic mechanism, to counteract chronic ischemia it is necessary to act on the hemorheological property of the blood flow, the latter largely determined by red blood cells, and to neutralize the oxidative stress due to the rebound effect of the resolution of the same ischemia; there is also a need to enhance the scavenger properties of the affected bone tissue [13,14,15].

Pentoxifylline (PTX) is a methylxanthine derivate that was originally approved by the Food and Drug Administration (FDA) to treat peripheral artery diseases and has been used to treat complications related to fibrosis for over 20 years [16,17,18]. PTX increases vasodilation and erythrocyte flexibility, and reduces blood viscosity which leads to an improvement in peripheral blood flow [17,18,19,20]. Tocopherols are a class of organic chemical compound consisting of various methylated phenols with beneficial effects. Tocopherols decrease tissue fibrosis and reduce inflammation, and they have antioxidant effects that protect cell membranes from lipid peroxidation by reducing the free radical damage generated during oxidative stress [17,18,21]. Pentoxifylline in combination with tocopherol is described as “PENTO protocol” for the management of osteoradionecrosis with beneficial antioxidant and antifibrotic properties [17]. Currently, these drugs are considered as having a positive synergistic therapeutic efficacy in treating chronic ischemic pathological conditions, such as diabetic microangiopathy, osteoradionecrosis, MRONJ, and osteomyelitis; however, the evidence is still limited and the mechanism of action remains unclear [17,18,21,22,23,24,25].

The aim of this randomized clinical study was to evaluate the effect of pentoxifylline and tocopherol in patients that had developed MRONJ after tooth extractions. The main objective was to demonstrate the outcomes of the pharmacological preparation with pentoxifylline and tocopherol in the management of medication-related osteonecrosis of the jaw.

## 2. Material and Methods

### 2.1. Study Design 

This study was planned as a prospective randomized controlled clinical study (RCT) and the randomization list was prepared using an online tool (http://graphpad.com/quickcalcs/randomize1.cfm (accessed on 15 July 2013)). Allocation concealment was ensured by double-blinding (the person who generated the randomization list and assigned the subjects to the groups was not involved in evaluating eligibility of individuals and their enrolment). This study was conducted between August 2013 and November 2017 at the Department of Oral Surgery and Maxillofacial Surgery, of the Francesco Miulli Regional Hospital, University of Bari (Bari, Italy). The follow-up period for 9 years until January 2023 was conducted at Oral Med Care srl, Regional Dental Medical Centre of Oral Surgery and Maxillo-Facial Surgery (Bitonto, Italy) that has an agreement with the University of Bari. The study protocol was approved by the Institutional Review Board of the Francesco Miulli Regional Hospital. (Number and date of the protocol: 203077—13 February 2014). This study was compliant with the principles set out in the Declaration of Helsinki on medical protocol and ethics. The trial was registered in the ISRCTN registry with study registration number ISRCTN18375975 (https://www.isrctn.com/ISRCTN18375975 (accessed on 10 July 2013)).

The patients included in the study were informed about the details of surgical treatment plan with the aims, treatment time, advantages, disadvantages, risks, and possible complications. All participants signed informed consent form for participation. 

### 2.2. Patient Selection

This clinical study included patients with MRONJ that were treated with a pharmacological preparation for two months with pentoxifylline and tocopherol before marginal sequestrectomy as surgical treatment for the management of the osteonecrosis lesions following tooth extractions. Null hypothesis was that the application of the pharmacological protocol had no effect on MRONJ. 

Inclusion criteria were:

- ONJ Stage I patients according to the staging criteria of the AAOMS of 2022 [6];
- Patients that needed surgical removal of the osteonecrosis lesion;
- Intake of BPs for over a period of 3 years for osteoporotic disease;
- Patients with ONJ lesion that developed after tooth/teeth extractions.

Exclusion criteria were:

- ONJ patients with Stage II and III according to the staging criteria of the AAOMS of 2022 [6];
- Any stage of ONJ related to any other combination or/and type of anti-resorptive other than BPs, such as anti-Rank-L (denosumab), antiangiogenic medications, and any other drug imputed for the development of MRONJ;
- Cancer patients with ONJ;
- Any stage of ONJ related to the association of antiresorptive with or without the combination of other drugs used for the development of MRONJ for the treatment of other patients with dysmetabolic, oncological, and metastatic bone diseases.

Patients treated with denosumab were excluded, because this drug does not accumulate in the bones as bisphosphonates, due to its different pharmacokinetics (in fact, being a monoclonal antibody, its half-life is 28 days given the hepatic clearance and in six months it disappears completely without having any residual effect of activity). 

Oncological patients were excluded as the general conditions do not allow waiting 2 months for the pharmacological preparation and the following 6 months for recovery, especially if the bone metastatic disease is progressive and the antiresorptive drug must be applied immediately. 

### 2.3. Randomization

Test group: patients allocated to this group were prescribed drug therapy for two months with pentoxifylline 600 mg (twice a day) and tocopherol 800 I.U. (once a day), before the surgical procedure of marginal sequestrectomy of the necrotic bone. Control group: patients allocated to this group had sequestrectomy operations without any pharmacological preparation. 

### 2.4. Pre-Operative Protocol: Diagnosis and Staging 

For all patients included in this study, the diagnostic process was completed by means of anamnesis, physical examination, and routine blood tests for surgical risk assessment, performed by the same analysis laboratory. These tests included: complete blood count (CBC), plasma proteins and protein electrophoresis, erythrocyte sedimentation rate (VES), COVID-19 test (PCR), prothrombin time (PT), partial thromboplastin time (PTT), international normalized ratio (INR), fibrinogen, antithrombin III, creatinine test, azotemia, sodium (Na), potassium (K), chloride (CL), estimated glomerular filtration rate (eGFR), uric acid, alanine transaminase (ALT), aspartate transferase (AST), GGT, total and fractionated bilirubin, and diagnostic information by means of orthopantomographic examination of the dental arches (orthopantomography) and cone beam computed tomographic (CBCT) examination of the maxillofacial region. 

All these patients were under a bisphosphonate drug and were classified as maxillary and mandibular MRONJ Stage I. None of the patients had drug holiday before interventions. 

The patients were randomly divided into two groups. In the test group (N = 108 test), each patient before surgery was subjected to pharmacological treatment with pentoxifylline (600 mg × 2/day) and tocopherol (800 I.U. × 1/day), for two months. After two months, a clear separation between necrotic and vital bone was assessed by means of diagnostic imaging (CBCT and MRI). Once this separation was confirmed, maxillary and mandibular marginal sequestrectomy was performed. The patients of the control group (n = 95), on the other hand, were immediately subjected to surgery. 

After surgery, the patients of the test group continued taking pentoxifylline and tocopherol (at the same dose as the pre-surgery period) for 6 months, to support bone healing. The control group patients, in addition to the standard oral hygiene protocol, were prescribed to rinse with sodium bicarbonate (1.5 g dissolved in 20 mL of physiological solution) twice daily for two months.

### 2.5. Standard Pharmacological Protocol

All the subjects received an oral hygiene program according to the full mouth disinfection protocol, with non-surgical periodontal treatment for each dental arch and mouth rinses with 0.2% chlorhexidine twice a day for seven days. On the day of surgery, antibiotic prophylaxis with amoxicillin and clavulanic acid (875 mg + 125 mg) was administered starting one hour before the surgical procedure and was continued as 2 tablets per day for a total of 7 days. Additional post-operative medications: metronidazole 250 mg tablet, 2 tablets every 12 h for 7 days, analgesics and non-steroidal anti-inflammatory drugs when needed. Patients were instructed to consume a semi-liquid diet for 7 days and were advised to avoid smoking. 

### 2.6. Surgical Protocol 

The operation was performed by the same surgeon (GC), always applying the same technique and the same antibiotic prophylaxis protocol. In brief, after local anesthesia with 4% articaine without vasoconstrictor, the surgery started with a marginal incision and elevation of a mucoperiosteal flap; the self-sequestered necrotic focus was exposed and removed. The sequestrectomy of the lesion was performed under hemostatic control. Debridement of the granulation tissue was made using ultrasonic instruments with rounding of the sharp bone edges. Intact bony walls were maintained with proper bleeding that was observable. Then, the wound was closed using resorbable sutures (4/0 Vycryl suture).

### 2.7. Follow-Up Evaluation

In both groups, the patients were monitored with regular checks at 7 days, 15 days, 1 month, monthly up to 6 months, and at 12 months after surgery. In the checks at 7 and 15 days, mucosal healing and any early post-operative complications were noted. In the 1- and 3-month checkups, any late post-operative complications were recorded. During the follow-up at 6 months after surgery, clinical and radiographic evaluation was performed, and bone healing was assessed. Mucosal healing without bone exposure within one month of surgery was a preliminary sign of success. Stability of this result over time, in addition to bone healing at 6 and 12 month assessment after surgery, confirmed case success. Taking AAOMS classification of 2022 as reference [6], all cases of non-mucosal healing, no bone healing, new bone exposure site, variation of stage towards worse, and relapse of lesions were classified as failures. 

### 2.8. Study Variables

The primary outcomes were the healing of the mucosa, assessed by absence of inflammatory signs at clinical inspection; the healing of the bone lesion, evaluated both radiologically (using cone-beam computed tomography, CBCT) and clinically, in a follow-up of at least 6 months after intervention; and the incidence of relapses.

Secondary outcome was the incidence of any post-surgical complication (other than ONJ relapse).

### 2.9. Data Collection 

Data collection included age, gender, type of bisphosphonate, route of administration, dosage, duration of treatment, outcomes of the treatment, complications, management of complications, mucosal healing, bone healing, MRONJ staging, and failures (relapses) for each patient. 

### 2.10. Statistical Analysis 

Continuous data were expressed as mean value ± standard deviation (SD), and discrete data as relative or absolute frequencies. The ranges of values were also reported, when appropriate. The normality of distributions of quantitative data was checked using the D’Agostino and Pearson omnibus normality test. If the data did not follow a normal distribution, median and 95% confidence intervals were used. The comparisons of outcomes between the two groups were made using parametric or non-parametric tests (Student’s *t*-test or Mann–Whitney test, respectively), as appropriate, for continuous data. Pearson’s chi squared test or Fisher’s exact test (when frequency was less than 5 in a group) was used for comparing the frequencies. The statistical analysis was performed using Stata 17 (StataCorp LLC, College Station, TX, USA). A *p*-value of 0.05 was considered as the significance threshold. 

## 3. Results

The initial population included a total of 697 female osteoporosis patients followed from August 2013 to January 2023. Of these, 377 patients had developed MRONJ following the intake of antiresorptive drugs for longer than 5 years, for disease management. Out of these, 202 patients developed ONJ after tooth extraction and were included in the study. All these patients were under bisphosphonate drug treatments for osteoporotic purposes and were classified as maxillary or mandibular MRONJ Stage I. 

In more detail, the study population consisted of 202 female patients (108 test, 94 control) with an average age of 66.4 ± 8.3 (SD) years (range 41 to 86 years). There were 21 patients with smoking habits (13 in the test group). All patients were scheduled as MRONJ Stage I and there was no difference in staging of the patients except one patient in control group that developed MRONJ Stage II. All were osteoporosis patients (mean T score −3.0 ± 0.4 range −2.4 to −4.6); there were no patients that were receiving bisphosphonate medications for oncologic reasons. One hundred and seventeen patients were otherwise healthy, while 85 patients had at least one or more of the medical conditions listed below, with number of subjects indicated in parenthesis: hypertension (16), dyslipidemia (22), hypothyroidism (3), hypovitaminosis D (51), venous insufficiency (3), chronic cardiac insufficiency (8), knee arthrosis (2), atrial fibrillation (3), fibromyalgia (1), anxiety syndrome (3—one associated with insomnia), hemicrania (1), diabetes mellitus (12), gut disease (2), chronic obstructive broncopneumopathy (2), epilepsy (2), trigeminal neuralgia (1), hemorrhoid (1), gastro-esophagus reflux (1).

The types of bisphosphonate taken by the included patients were the following:

Amino-containing BPs: Fosamax© (alendronic acid) (MSD Italia S.r.l., Milano, Italy) (total 23, 12 in test); Fosavance© (alendronic acid and vitamin D) (MSD Italia S.r.l., Milano, Italy) (total 27, 14 in test); Binosto© (ibandronic acid) (Abiogen Pharma Spa, Pisa, Italy) (total 21, 11 in test); Adrovance© (alendronic acid and vitamin D) (AddendA Pharma S.r.l., Milano, Italy) (total 19, 9 in test); Optinate© (risendronic acid) (Theramex Italy, Milano, S.r.l., Italy) (total 13, 4 in test); Bonasol© (alendronic acid) (Bruno Farmaceutici Spa, Rome, Italy) (total 15, 8 in test (1 patient had additional Oxafort)); Adronat© (alendronic acid) (Neopharmed Gentili S.r.l., Milano, Italy) (total 14, 10 in test), Bonviva© (ibandronic acid) (BB Farma S.r.l., Samarate, Italy) (total 11, 9 in test); Alendronato© (alendronic acid) (Accord Healthcare Italia S.r.l., Milano, Italy) (total 6, 4 in test); Actonel© (total 26, 11 in test); Risedronato© (risendronic acid) (Accord Healthcare Italia S.r.l., Milano, Italy) (total 18, 9 in test); Ibandronato© (ibandronic acid) (Teva Italia Srl, Milano, Italy) (total 3, 2 in test); Nerixia© (neridronic acid) (Abiogen Pharma Spa, Pisa, Italy) (total 7, 5 in test). 

Non-amino BPs: Difosfonal© (clodronic acid) (SPA-Soc.Pro.Antibiotici, Milano, Italy) (total 11, 6 in test (all changed afterwards)); Clody© (clodronic acid) (Promedica Srl, Rome, Italy) (total 11, 7 in test (4 in test had additional Bonviva and 2 had additional risedronate); Clodronato© (clodronic acid) (Abc Farmaceutici Spa, Ivrea, Italy) (total 2, 1 in test); Niclod© (clodronic acid) (Savio Pharma Italia S.r.l., Pisa, Italy) (total 6, all in control); Clasteon© (clodronic acid) (Abiogen Pharma Spa, Pisa, Italy) (total 2, 2 in test—1 in test changed to Actonel afterwards). 

Other medications that were given to patients as an alternative: all these patients were started with BPs: Prolia© (Amgen Srl, Milano, Italy) (denosumab) (total 2, 2 in test) (all were patients that started with Fosamax© or Binosto© ( Abiogen Pharma Spa, Pisa, Italy) than changed to denosumab); Osseor© (Strider, Sesto San Giovanni, Italy) (strontium ranelate) (total 8, 3 in test); Oxafort© (Shedir Pharma Srl Unipersonale, Galdo-SantÀngelo, Italy) (integrator based on Ca, Vit D, Vit K) (total 8, 1 in test had additional Bonasol©); Rocatrol© (Roche, Monza, Italy) (integrator based on Ca, Vit D (2—all in control), Natecal D3© (calcium and vitamin D) (Italfarmaco Spa, Milan, Italy) (total 10, 5 in test).

Regarding the administration route of medications: in the test group at the beginning 93 patients had intra-oral BPs and 15 had intra-muscular BPs; in the control group at the beginning 80 patients had intra-oral BPs and 14 had intra-muscular BPs. All patients taking intra-muscular administration changed the route to intra-oral within one year. 

Table 1 summarizes the main local features of the patients and the main outcomes of the study. Comparisons between groups were performed using Pearson’s chi square or Fisher’s exact test, as appropriate. Significance is reported in the last column.

Healing of the mucosa at one month was observed in all test group patients, while in 17% of subjects in the control group (16 patients) the mucosa did not heal (*p* < 0.0001). The incidence of relapse of the lesions after initial healing was less than 1% in the test group (1 patient) and it was 71% in the control group (67 patients). All relapses occurred within 6 months. In total, only one failure was observed in the test group and 83 (88%) in the control (*p* < 0.0001). Smoking habits did not affect either relapse (*p* = 0.31) or healing (*p* = 0.77).

During the follow-up, seven complications (7.4%) were seen in patients in the control group in addition to failure (three abscesses with cutaneous fistula, two mucous fistula, two phlegmon), while none of the subjects in the test group experienced any complications.

In cases of complications, the following treatment was applied: Rocefin (ROCHE SPA) flacon 2 mg intra-vascular (i.v.) (1 flacon in 100 mL of serum physiological) for 7 days, in association with metronidazole 500 mg i.v. (TEOFARMA SRL) (1 flacon every 12 h for 7 days), levoxacin 500 mg i.v. (GLAXOSMITHKLINE SPA) (1 flacon in 100 mL of serum physiological) for 7 days. 

After 6 months, all the patients of the control group with failures or complications were managed as the patients of the test group with pentoxifylline and tocopherol, at the same dosage. A second surgery was performed when needed. Then, for all the lesions of control patients healing was achieved. All patients were then monitored every six months during the follow-up (mean follow-up period of 7.8 ± 0.3 years, range 7.3 to 8.3 years), during which no patient experienced any relapse or additional problems. 

**Figure 1:**
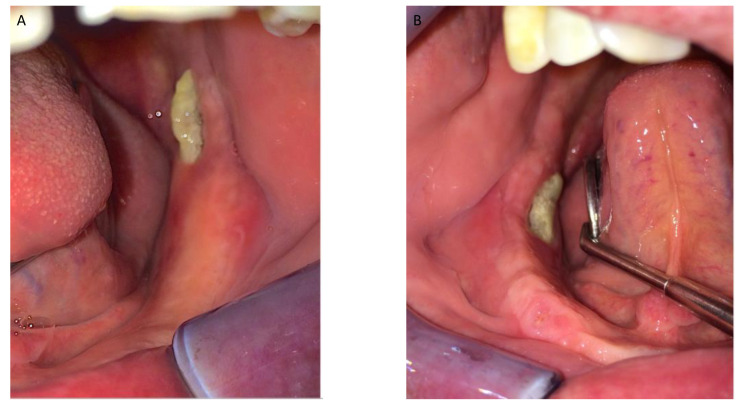
Pre-operative intra-oral view from a MRONJ patient with bilateral defect. (**A**) left side; (**B**) right side.

**Figure 2:**
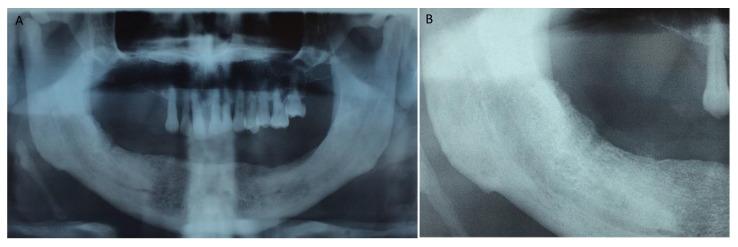
Pre-operative orthopantomography of the same MRONJ patient showing bilateral defect; (**A**) shows the panoramic view; (**B**) shows more detail of the right side of the mandible.

**Figure 3:**
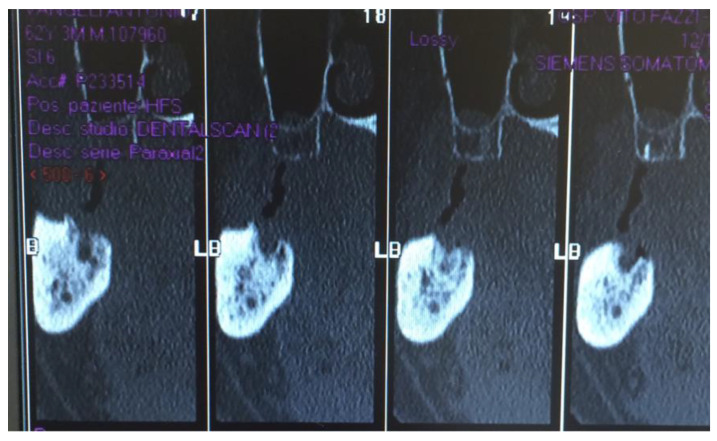
Pre-operative CBCT of the MRONJ patient, showing a reduction in the medullar space.

**Figure 4:**
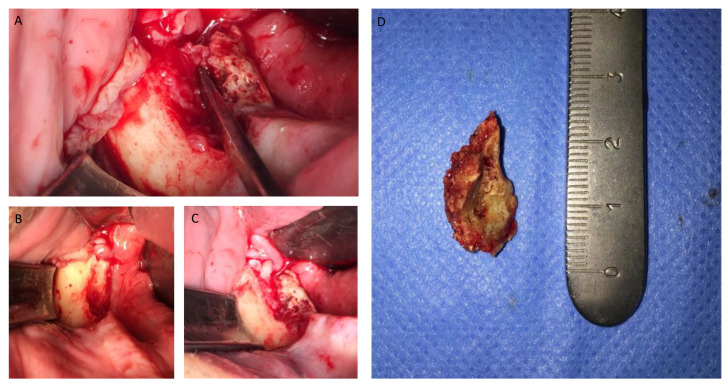
(**A**–**D**) Intra-operative view showing operation steps and resected osteonecrotic tissue from the right side of the mandible. After pharmacological preparation with pentoxifylline and tocopherol, the cleavage plane created by the reactive granulation tissue can be clearly seen which tends to separate the necrotic tissue from the healthy bone. Sequestrectomy was made using an osteotome to obtain the necrotic specimen. After the debridement, the bottom of the surgical area was bleeding properly, which creates a favorable environment for healing.

**Figure 5:**
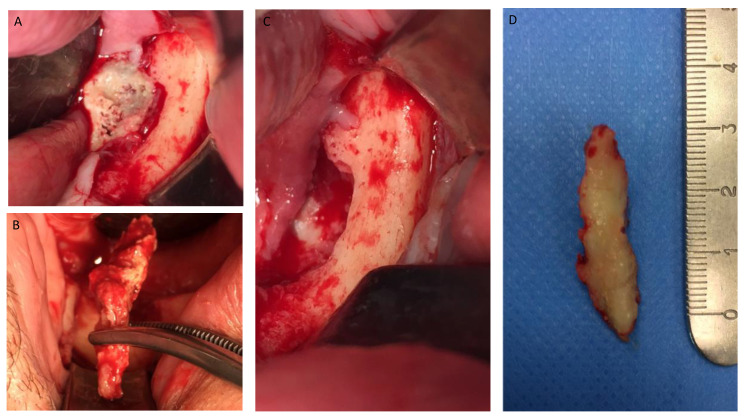
(**A**–**D**) Intra-operative view showing sequestrectomy operation and resected osteonecrotic tissue from the left side of the mandible.

**Figure 6:**
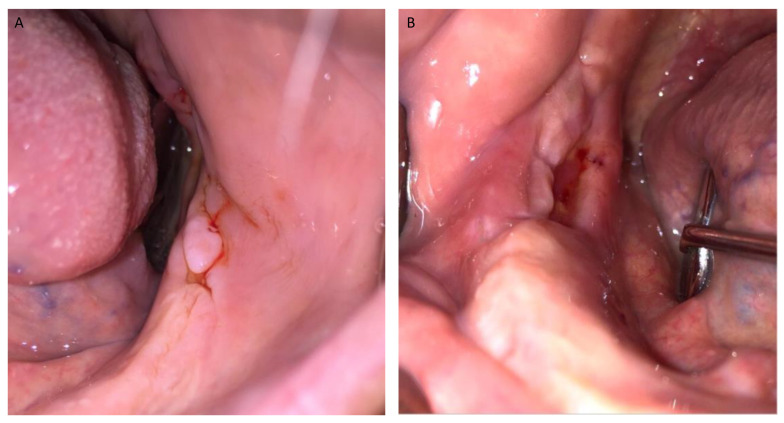
(**A**,**B**) Post-operative intra-oral view at 2 week follow-up. Mucosal healing without bone exposure can be seen.

**Figure 7:**
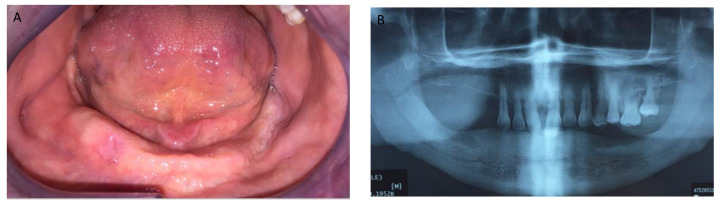
(**A**,**B**) Post-operative intra-oral and radiographic view at 6 month follow-up showing healed soft (**A**) and hard (**B**) tissues.

Figure 1, Figure 2, Figure 3, Figure 4, Figure 5, Figure 6 and Figure 7 show the progress of treatment in one of the patients of the test group. In Figure 3 reduction of the medullar space is visible, that may create a chronic ischemic area susceptible to necrosis.

## 4. Discussion

In patients under treatment with BPs, tooth extractions are avoided in the clinics as much as possible since they are considered a major risk factor for MRONJ. However, MRONJ can also develop from a tooth that is an infection source [26]. In cases of MRONJ, as the classical approach in the past, sequestrectomy was performed immediately, thinking that at the basis of the pathogenesis there was only the bacterial infection capable of triggering necrosis. In MRONJ lesions, antibacterial therapy is not sufficient by itself for the control of the infection and healing. Additionally, it is necessary to remove the necrotic bone from the healthy tissues. The high recurrence rates observed in the control group suggested that infection alone is not sufficient to cause ONJ and that its eventual eradication does not prevent recurrence [27].

Today, necrotic bone infection is considered only the cause of extension and aggravation of the clinical picture [28]. Furthermore, the association of ONJ with antiangiogenic drugs has been ascertained as fact and these drugs can exacerbate ONJ that shows no evidence of necrotic bone infection. This demonstrates that the pathogenetic mechanism, on which osteonecrosis can develop (also common with anti-resorptive drugs) is the result of chronic ischemia that develops after their use [29].

The antiangiogenics block the formation of new vessels or angiogenesis, while the antiresorptive drugs significantly increase the bone density to the detriment of the marrow spaces and reduce the vascularization of the jaws, thereby creating chronic ischemic conditions, which make the bone tissue susceptible to infections, with the possibility of developing osteonecrosis [14]. Based on this pathogenetic evidence, various therapies have been described in the literature for the management of MRONJ [30,31,32,33] among which pentoxifylline and tocopherol were reported to have successful outcomes in a very limited number of reports [16,17]. 

From the analysis of the scientific literature on the management of cases of MRONJ Stage I, in both oncological and non-cancer patients, demolitive surgery represents the first therapeutic choice. This choice involves wide resections, to obtain bleeding bone margins or stumps, a sign of vitality and a necessary condition for the healing process and the absence of recurrences. Very often, to achieve these objectives, the resections become total or sub-total, in the latter case creating structural anatomical conditions where the risk of pathological fracture becomes high. In both cases bone reconstruction procedures are planned, also with microsurgical methods with executions of free flaps (e.g., free flap fibula). The high morbidity of the procedures described above and the considerable biological cost on the part of the patient are immediately obvious. Their high percentage of failure, the impossibility of repetitive procedures, and the notable negative impact on the functions of the entire stomatognathic system have prompted research to associate surgery with adjuvant treatments that can help to overcome the problems described above (such as ozone therapy, hyperbaric therapy with high oxygen rates, surgery with the Nd-YAG Laser, the aid of the VELscope© to predictably define the border between vital and necrotic bone, and the application of blood derivatives and components) [34,35,36,37]. All these options share the goal of avoiding or reducing the extent of surgical demolition, supporting the healing process, and reducing recurrence rates. However, the analysis of the scientific literature on the use of these methods does not clearly and unequivocally define the real adjuvant impact in the management of MRONJ among them [34,35,36,37]. Very often it has proved useless and not very effective due to the high rate of oxygen free radicals which caused considerable damage to the vital residual bone component, producing the paradoxical effect of increasing necrosis itself. Laser surgery, apart from a minor impact on procedural morbidity, has no contrasting effect on the etiopathogenesis of ONJ; the same applies to surgery with the aid of the VELscope© [34]. Furthermore, many studies, while showing promising results, are conditioned by the small number of patients treated, not allowing a robust statistical analysis with impact to facilitate and develop guidelines. This problem also afflicts the studies that have proposed pentoxifylline and tocopherol as an adjuvant method in the surgical management of MRONJ [37,38,39,40].

This study reports an adjuvant method to the surgical management of MRONJ Stage I with an objective to affect the progress of the disease by improving blood circulation, by making the surgery as minimally invasive as possible and avoiding excessively destructive procedures. Consequently, the quality of life of patients can improve. The application of the pharmacological preparation protocol before surgery, using pentoxifylline and tocopherol, aims to counteract the chronic ischemic condition and overcome the oxidative stress associated with the rebound effect by improving blood circulation. The goal is to create ideal bone microenvironmental conditions for the healing process. This increase in blood flow can bring the entire inflammatory and immune cellular compartment at the infected site, by isolating the necrotic bone tissue from the vital and reactive bone tissue. As a result, this granulation tissue will create a cleavage plane, which makes the sequestrectomy operation less aggressive than the past. Due to the blood circulation, enhanced by the pharmacological protocol, both mucosal and bone healing were achieved predictably. Lastly, the absence of relapse in the test group during the follow-up can be justified by the accelerated healing. In the control group, the recurrence rate was higher than the test group. This unhealed chronic ischemic clinical condition at the affected site might occur because of the oxidative stress, the lack of inflammatory and immune reactive capacity of the bone, and poor blood circulation. Furthermore, sequestrectomy surgery itself is a destructive operation and can constitute a new trigger for the development of osteonecrosis as it aggravates the ischemic condition to the neighboring tissues. 

According to the results of this study, patients in the test group experienced no relapse and no complications during six months after surgery. On the other hand, in the control group, 71% of the patients relapsed immediately or within two months, and the remainder did not heal, or developed infectious complications such as the abscess or phlegmon type. After 6 months, the patients in the control group received the same pharmacological protocol as the patients in the test group; consequently, MRONJ lesions completely resolved clinically, without experiencing any relapse or additional problems in the follow-up. The importance of this study is that the pre-operative pharmacological preparation that is used acts by actively counteracting the etiological factors which are at the pathogenetic basis of osteonecrosis. Pentoxifylline counteracts the chronic ischemic condition which underlies the development of osteonecrotic tissue, allowing the suffering bone to completely recover back to its vitality and to sequester the necrotic bone simultaneously. Additional tocopherol eliminates the toxic aspects of oxygen free radicals due to the rebound effect of the restoration of an adequate blood flow of the bone. Furthermore, its use creates microenvironmental conditions for the adequate development of new blood vessels, for local stimulus effect on angiogenesis. 

In the literature, there are very limited reports on pentoxifylline and tocopherol combinations as adjunctive therapy in the management of osteonecrotic tissues, especially in cases of MRONJ [17,37,38,39,40]. This clinical research on 202 MRONJ patients can contribute to the literature due to the relatively large number of subjects and long follow-up period of around 8 years. However, it also has some limitations. One of the limitations of this work is that no oncologic patients were included, and it would be interesting to evaluate and compare their findings with osteoporotic patients. Furthermore, all the patients had osteonecrosis with Stage I, excluding patients with more advanced stages. In Stage I patients only the alveolar process of the maxilla and mandible is affected, while in Stage II, in addition to the presence of infection and inflammation, the necrotic process extends beyond the alveolar process to the basal bone of the jaws. This would imply a variation of the surgical procedure in which not only a demolitive but also a reconstructive approach would be required. Therefore, the surgical results would have been difficult to interpret, due to the introduction of further variables. In osteoporotic patients, Stage II is much less frequent than Stage I, because the early diagnosis of the problem is faster, and usually the general conditions of the patient are better than in cancer patients. In fact, in the latter, since the general conditions are poorer, the doses of antiresorptives are higher, and the route of administration is different (usually intravenous), the MRONJ onset is already in advanced stages. Additionally, the residual presence of bisphosphonate should be investigated by comparing the chemical composition of the sequestrated osteonecrotic bone before and after drug preparation in a subsequent study. Obviously, these limitations represent new research objectives for future studies and this report should be considered as preliminary research on this topic, as it represents a first step towards the goal of developing a valid non-aggressive protocol for the management of MRONJ patients.

## 5. Conclusions

According to the results of this clinical study, the administration of pentoxifylline and tocopherol from two months before sequestrectomy, and up to 6 months during the bone healing period, seems to be beneficial for the resolution of MRONJ.

## Figures and Tables

**Table 1:** Information about intra-oral situation of the patients and statistical comparison between the groups.

		Patients	Test	Control	*p*-Value
	Total patients	202	108	94	
Location of the lesion (arch)	Maxilla Mandible	69	42	27	0.13
		133	66	67	
Distribution according to bone density in maxilla	D1 D2 D3	0	0	0	0.43
		50	29	21	
		19	13	6	
Distribution according to bone density in mandible	D1 D2 D3	60	36	24	0.03
		73	30	43	
		0	0	0	
Location according to quadrant	Quadrant 1 Quadrant 2 Quadrant 3 Quadrant 4 Q3—Q4	44	29	15	0.26 *
		26	14	12	
		72	38	34	
		55	24	31	
		6	4	2	
Type of extraction	Single Multiple	70	31	19	0.68
		67	44	23	
	Cyst	7	4	3	0.85 *
Implant removal	Yes No	30	15	15	0.68
		172	93	79	
Incidence of relapse	Yes No	68	1	67	<0.0001 *
		134	107	27	
Incidence of healing	Yes No	186	108	78	<0.0001 *
		16	0	16	
Total failures	Yes No	84	1	83	<0.0001 *
		118	107	11	
Incidence of complications	Yes No	7	0	7	0.0004 *
		195	108	87	

Quadrant: the division of the jaws into 4 quadrants. Quadrant 1 represents right maxilla, Quadrant 2 represents left maxilla, Quadrant 3 represents left mandible, Quadrant 4 represents right mandible. D: Density of bone. *: Fisher exact test.

## Data Availability

Data of this work is available upon request.
